# A minimally invasive and safe surgical approach to resect anterior superior sulcus tumors

**DOI:** 10.1016/j.ijscr.2020.02.047

**Published:** 2020-02-28

**Authors:** Soichi Oka, Kenji Ono, Kenta Kajiyam, Katsuma Yoshimatsu

**Affiliations:** Thoracic Surgery, Kokura Memorial Hospital, Kitakyushu-shi, Japan

**Keywords:** SST, superior sulcus tumor, CT, computed tomography, VATS, video-assisted thoracic surgery, FDG, fluorodeoxyglucose, superior sulcus tumor, Pancoast tumor, Lung cancer, Video-assisted thoracic surgery

## Abstract

**Introduction:**

Superior sulcus tumors (SSTs) are a wide range of tumors invading a section of the apical chest wall called the thoracic inlet. The unique characteristics of SSTs lie in the anatomy of the region where these tumors occur. For this reason, a surgical approach to treating these tumors is technically demanding, and complete resection may be difficult to accomplish.

**Case presentation:**

A 71-year-old Japanese man presented at our hospital due to left anterior chest pain and an abnormal chest CT scan showing a 40 × 33 × 30-mm tumor located in the left anterior apex of the thoracic inlet. This tumor had invaded the first and second rib and was located near the subclavian vein. There was no significant distant metastasis. Therefore, we performed surgical resection. The surgical procedure included three steps. First, we performed VATS observation via the left thoracic cavity. Second, via the transmanubrial approach, we obtained tumor-free margins of the anterior cervical structures. Third, through VATS in the left lateral decubitus position, we performed left upper lobectomy and mediastinal lymph node dissection. This surgery was successful, with no postoperative complications.

**Disucussion:**

This surgical approach was effective and safe for treating a superior sulcus tumor located the anterior apex of the thoracic inlet. Next, VATS lobectomy is minimally invasive and safe after the transmanubrial approach for managing anterior superior sulcus tumor.

**Conclusion:**

We experienced a case of locally advanced superior sulcus tumor located at the anterior apex of the thoracic inlet and performed complete resection.

## Highlights

• A surgical approach for SST to treating these tumors is technically demanding, and complete resection may be difficult to accomplish.
• We experienced a case of locally advanced superior sulcus tumor located at the anterior apex of the thoracic inlet and performed complete resection.
• This surgical approach (VATS observation and transmanubrial approach) was effective and safe.
• VATS lobectomy is minimally invasive and safe after the transmanubrial approach.

## 1 Introduction

Superior sulcus tumors are a wide range of tumors invading an area of the apical chest wall called the thoracic inlet. The unique characteristics of superior sulcus tumors lie in the anatomy of region where these tumors occur. For this reason, a surgical approach to treating these tumors is technically demanding, and complete resection may be difficult to accomplish [1]. The treatment of superior sulcus tumor has evolved greatly over the years; initially thought to be inoperable, the first case of surgical removal was reported in 1956 by Chardack and MacCallum [2]. In the 1990s, induction chemoradiotherapy followed by radical surgical resection was introduced as a new standard treatment for superior sulcus tumors. This treatment brought in improved outcomes and remains the gold standard today [1].

We experienced a case of a locally advanced superior sulcus tumor located at the anterior apex of the thoracic inlet and performed complete resection. This work has been reported in line with the SCARE criteria [3].

## 2 Case presentation

**Fig. 1:**
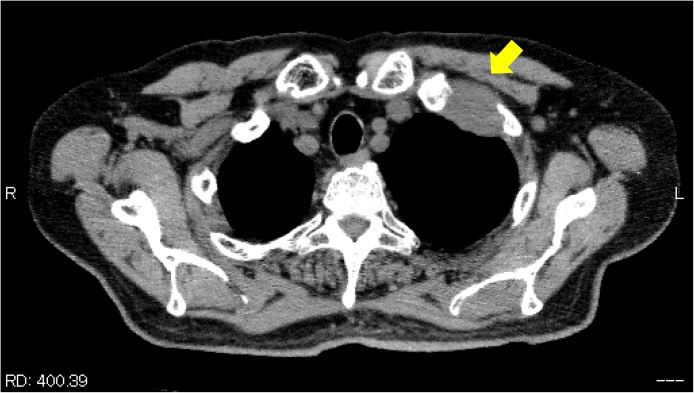
Computed tomography of the chest showing the localization of this tumor. This tumor was located at the left apex thoracic inlet and involved the first and second ribs.

A 71-year-old Japanese man presented at our hospital due to left anterior chest pain and an abnormal chest computed tomography (CT) scan showing a 40 × 33 × 30-mm tumor located at the left anterior apex of the thoracic inlet. This tumor had invaded the first and second rib and was located near the subclavian vein (Fig. 1). This patient has chronic renal failure and is undergoing peritoneal dialysis.

**Fig. 2:**
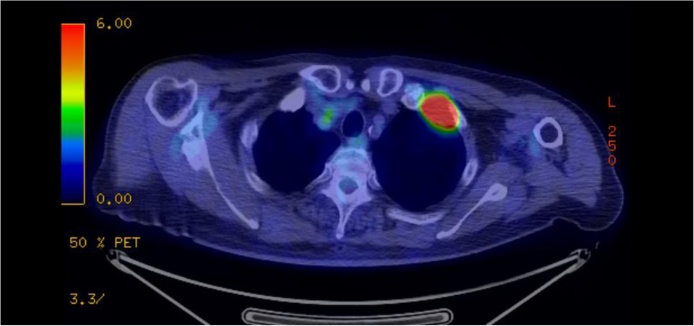
18-fluorodeoxyglucose (FDG) positron emission tomography/computed tomography showed a high FDG uptake within the tumor (SUV_max_: 12).

We performed bronchoscopy to make a definitive diagnosis but were unable to obtain a diagnosis. 18-fluorodeoxyglucose (FDG) positron emission tomography/computed tomography showed an increase standard uptake value in the tumor (Fig. 2). There was no significant distant metastasis. We did not performe neoadjuvant therapy because we determined that this tumor was resectable. Therefore, we performed surgical resection for this superior sulcus tumor located at the anterior apex of the thoracic inlet because the tumor was suspected to be local invasive lung cancer.

**Fig. 3:**
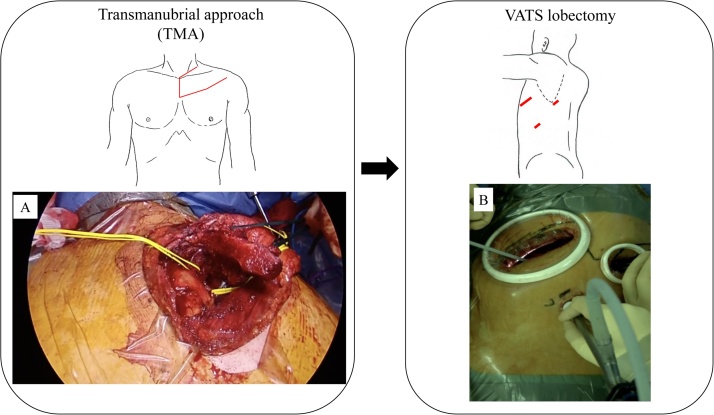
Diagram of the surgery and surgical view. We performed the transmanubrial approach. The tumor was located near the subclavian vessels, and we were able to remove the tumor from these major vessels safely. (A). VATS in the left lateral decubitus position, through which we performed left upper lobectomy and mediastinal lymph node dissection (B).

The surgical procedure included three steps. The diagram of the surgery and surgical view are shown in Fig. 3. First, we performed VATS (Video-assisted thoracic surgery) exploration via the left thoracic cavity. We then confirmed the resectability and lack of dissemination. Second, via the transmanubrial approach, we obtained tumor-free margins of the anterior cervical structures. The tumor was located near the subclavian vessels, and we were able to remove the tumor from these major vessels safely. We then disconnected the anterior first and second ribs using a wire saw. We were able to cut off the tumor invading anterior chest wall before closing the anterior wound. After that, we reconstructed the anterior chest wall using Gore-Tex Dual Mesh (Japan Gore-tex Inc., Tokyo, Japan) and closed the anterior wound. Third, through VATS in the left lateral decubitus position, we performed left upper lobectomy and mediastinal lymph node dissection. The operative time was 8 h 7 min, and the amount of intraoperative bleeding was 580 ml.

Pathologically, complete resection was achieved. The pathological diagnosis was stage IIB squamous cell carcinoma (p-T3N0M0). The patient was discharged from our hospital 13 days after surgery. This surgery was successful, with no postoperative complications.

## 3 Discussion

Two points should be noted in association with this case. First, this surgical approach (VATS exploration and transmanubrial approach) was effective and safe for treating a superior sulcus tumor located at the anterior apex of the thoracic inlet. We first performed VATS via the left thoracic cavity. Rosso et al. reported that “VATS observation first” is useful for excluding previously undetected pleural dissemination and for precisely defining the tumor location [7]. We therefore agree with Rosso’s “VATS observation first” approach. The lesion in this patient was suspected of invasion the subclavian vessels. Therefore, the transmanubrial approach was useful for removing the tumor from major vessels. We were fortunately able to remove the tumor from the subclavian vessels without issue; however, if we had not been able to remove it, we could alternatively have resected and reconstructed the subclavian artery via the transmanubrial approach. Indeed, we previously reported several aggressive surgeries in which we used the transmanubrial approach and resected/reconstructed major vessels [[4], [5], [6]].

Second, VATS lobectomy is minimally invasive and safe after the transmanubrial approach for managing anterior superior sulcus tumor. We performed left upper lobectomy and mediastinal lymph node dissection through VATS in the left lateral decubitus position after adopting the transmanubrial approach. Given the visual difficulty of performing lobectomy via the transmanubrial approach, it is necessary to change the position to the lateral decubitus position. However, thoracotomy is highly invasive. Therefore, VATS lobectomy is a good approach. As in the present case, VATS lobectomy can be performed if the tumor-invaded area of the anterior chest wall can be cut off via the transmanubrial approach. The present patient experienced little pain, and the postoperative course was good.

## 4 Conclusion

We experienced a case of locally advanced superior sulcus tumor located at the anterior apex of the thoracic inlet and performed complete resection.

## Funding

We have no sources of funding for our research.

## Ethical approval

We got ethical approval from ethical committee of Kokura memorial hospital, Japan.

## Consent

We had informed consent from this patient for writing this paper.

## Author contribution

Soichi Oka; study design, writing. Kenji Ono; study design, other. Kenta Kajiyama; other. Katsuma Yoshimatsu; other.

## Registration of research studies

My research registry number is 1565.

## Guarantor

Soichi Oka and Kenji Ono.

## Provenance and peer review

Not commissioned, externally peer-reviewed.

## Declaration of Competing Interest

We have no conflicts of interest.

## References

[1] Marulli G., Battistella L., Mammana M., Calabrese F., Rea F. (2016). Superior sulcus tumors (Pancoast tumors). Ann. Transl. Med..

[2] Chardack W.M., Maccallum J.D. (1953). Pancoast syndrome due to bronchiogenic carcinoma: successful surgical removal and postoperative irradiation; a case report. J. Thorac. Surg..

[3] Agha R.A., Borrelli M.R., Farwana R., Fowler A., Orgill D.P., For the SCARE Group (2018). The SCARE 2018 statement: updating consensus surgical case report (SCARE) guidelines. Int. J. Surg..

[4] Oka S., Matsumiya H., Shinohara S. (2016). Total vertebrectomy (Th2) and dissection of the subclavian artery for a superior sulcus tumor invading the spine: a case report. Int. J. Surg. Case Rep..

[5] Oka S., Taira A., Shinohara S. (2016). Complete resection of thymic sarcomatoid carcinoma through total arch replacement. Ann. Thorac. Cardiovasc. Surg..

[6] Oka S., Kobayashi K., Matsumiya H. (2017). An effective and safe surgical approach for a superior sulcus tumor: a case report. Int. J. Surg. Case Rep..

[7] Rosso L., Palleschi A., Mendogni P., Nosotti M. (2016). Video-assisted pulmonary lobectomy combined with transmanubrial approach for anterior Pancoast tumor resection: case report. J. Cardiothorac. Surg..

